# DFT Study of Single and Double Proton Transfer Mechanisms in Schiff Base Formation from 3-Pyridinecarboxaldehyde and Aminobenzoic Acid Isomers

**DOI:** 10.3390/molecules31122050

**Published:** 2026-06-11

**Authors:** Ion Arsene, Viorica Purcel, Andrei Rotaru

**Affiliations:** 1 Department of Chemistry, “Ion Creangă” State Pedagogical University of Chisinau, Str. Ion Creanga, Nr.1, 2069 Chisinau, Moldova; arsene.ion@upsc.md (I.A.); purcel.viorica@upsc.md (V.P.); 2 Department of Physical Chemistry and Quantum Chemistry, Institute of Chemistry of Chisinau, Moldova State University, Str. Academiei, Nr. 3, 2028 Chisinau, Moldova; 3 Department of Engineering Science, Babeş-Bolyai University, Str. Mihail Kogălniceanu, Nr. 1, 400084 Cluj-Napoca, Romania; 4 Department of Chemical Thermodynamics, Institute of Physical Chemistry–Ilie Murgulescu, Romanian Academy, Splaiul Independenţei, Nr. 202, 060021 Bucharest, Romania

**Keywords:** Schiff base, DFT calculations, proton transfer, reaction mechanism, transition state, activation energy

## Abstract

A comparative density functional theory (DFT) study was performed to elucidate the mechanistic details of Schiff base formation between 3-pyridinecarboxaldehyde and the three positional isomers of aminobenzoic acid (o-, m-, and p-). Both single proton transfer (SPT) and methanol-assisted double proton transfer (DPT) pathways were systematically investigated in the gas phase and within a polarizable continuum model (PCM) for methanol. All stationary points were optimized at the B3LYP/6-31G and 6-311++G(d,p) levels, and transition states were confirmed by vibrational frequency and intrinsic reaction coordinate (IRC) analyses. The results reveal that the DPT mechanism is consistently associated with significantly lower activation free energies compared to the direct SPT pathway, particularly in methanol, where solvent-mediated proton relay markedly stabilizes the transition states. The positional effect of the amino group influences both the electrostatic potential distribution and the activation barriers, with the para isomer exhibiting enhanced nucleophilicity and improved reaction efficiency. These findings provide detailed mechanistic insight into solvent-assisted proton transfer processes in Schiff base synthesis and highlight the cooperative role of hydrogen-bond networks in reducing energetic barriers.

## 1. Introduction

Schiff bases represent an important class of imine-containing compounds obtained through the condensation of primary amines with aldehydes or ketones. Owing to the presence of the azomethine (–C=N–) group, these compounds exhibit a broad spectrum of biological activities, including antibacterial, antifungal, antiviral, anticancer, and antioxidant effects [1,2,3,4,5,6,7,8]. In addition to their pharmacological relevance, Schiff bases play a central role in coordination chemistry, acting as versatile ligands capable of stabilizing transition metal complexes with applications in catalysis, gas storage, and functional materials [5,9,10,11,12,13,14,15,16,17].

Beyond their synthetic and applied importance, understanding the mechanistic aspects of Schiff base formation remains essential for improving reaction efficiency and selectivity. The condensation process involves a nucleophilic attack followed by proton transfer and dehydration steps, where proton migration constitutes a key mechanistic event [18,19,20,21,22]. Previous theoretical investigations have shown that the proton transfer may proceed either directly or through solvent-assisted pathways, significantly influencing activation energies and transition state stabilization [23,24,25,26].

Particular attention has been given to solvent effects, especially in protic media such as water or methanol, where hydrogen-bond networks can mediate double proton transfer (DPT) processes [27]. Such cooperative mechanisms often lead to substantial reductions in activation barriers compared to single proton transfer (SPT) routes [20,21,22,25,26]. Despite these advances, systematic comparative studies addressing the influence of positional isomerism on SPT versus DPT mechanisms remain limited.

Although related systems involving 4-pyridinecarboxaldehyde have been previously investigated [28,29], a comprehensive DFT analysis of proton transfer pathways in the condensation of 3-pyridinecarboxaldehyde with o-, m-, and p-aminobenzoic acids has not been thoroughly examined. In particular, the combined influence of electronic distribution, electrostatic potential, and solvent-assisted proton relay on activation barriers requires further clarification.

In this study, we present a detailed DFT investigation of single and double proton transfer mechanisms in the condensation reaction between 3-pyridinecarboxaldehyde and aminobenzoic acid isomers, both in the gas phase and in methanol solution. The objective is to elucidate the energetic, structural, and electronic factors governing reaction efficiency and to assess the cooperative role of solvent in facilitating proton transfer.

## 2. Computational Methods

All quantum chemical calculations were performed using the Gaussian program package [30]. Geometry optimizations of reactants, intermediates, transition states, and products were carried out within the framework of density functional theory (DFT) employing the B3LYP hybrid functional [31]. To assess the influence of basis set size on the calculated activation parameters, two basis sets were employed: the split-valence 6-31G basis set [32] and the extended triple-ζ 6-311++G(d,p) basis set [32]. The use of both basis sets allowed a comparative evaluation of the energetic sensitivity of single proton transfer (SPT) and double proton transfer (DPT) pathways to the level of electronic description.

Frequency calculations were performed at the same level of theory to characterize the nature of all stationary points. Minima were confirmed by the absence of imaginary frequencies, whereas transition states exhibited a single imaginary frequency corresponding to the proton transfer coordinate. Intrinsic reaction coordinate (IRC) calculations were carried out to verify the connectivity between transition states and their associated minima, following standard procedures for proton transfer reactions [33]. Zero-point energy (ZPE) corrections and thermal corrections were included in the thermochemical analysis. Activation parameters discussed in this work correspond to corrected energetic values obtained at 298.15 K and 1 atm.

Solvent effects were modeled using the polarizable continuum model (PCM) as implemented in Gaussian [34,35,36], with methanol chosen to approximate experimental protic conditions. The inclusion of solvent effects enables evaluation of dielectric stabilization and hydrogen-bond-assisted proton relay mechanisms, which are particularly relevant for DPT pathways [20,37]. All structures (reactants, intermediates, transition states, and products) were fully optimized in the presence of the solvent using PCM; thus, not only single-point energy calculations on gas-phase-optimized geometries were considered.

## 3. Results and Discussion

### 3.1. Electronic Effects and MEP Analysis

The geometric configuration of aminobenzoic acid isomers (ortho, meta, and para) directly influences their interaction with 3-pyridinecarboxaldehyde during the initial stage of the condensation reaction. The relative positions of the –NH_2_ and –COOH groups on the aromatic ring govern both the spatial orientation of the nucleophilic center and the possibility of intramolecular hydrogen bonding. These interactions may either stabilize specific conformations or reduce the electronic accessibility of reactive sites.

Thus, molecular geometry emerges as a decisive factor in determining reactivity. In the ortho isomer, the proximity between –NH_2_ and –COOH favors intramolecular hydrogen bonding, which restricts the optimal orientation of the nitrogen lone pair toward the carbonyl carbon. In the meta isomer, the absence of effective electronic cooperation between substituents leads to a more dispersed charge distribution and diminished localization of electron density on the amino group. In contrast, the para isomer exhibits greater spatial separation between substituents, allowing the amino group to adopt a more favorable orientation for nucleophilic attack.

**Figure 1:**
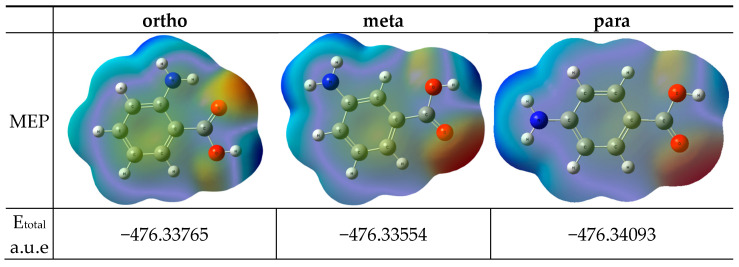
Molecular electrostatic potential (MEP) map calculated at the 6-311++G(d,p) level.

To quantify these electronic effects, molecular electrostatic potential (MEP) surfaces were analyzed at the B3LYP/6-311++G(d,p) level (Figure 1). The MEP maps provide a direct visualization of nucleophilic and electrophilic regions, enabling evaluation of the intrinsic nucleophilicity of the –NH_2_ group and its predisposition to interact with the aldehydic carbon.

The calculated electrostatic potential extrema are:

- o-aminobenzoic acid: MEP = −37.12/+37.12 kcal/mol;
- m-aminobenzoic acid: MEP = −39.19/+39.19 kcal/mol;
- p-aminobenzoic acid: MEP = −42.40/+42.40 kcal/mol.

As shown in Figure 1, the ortho isomer displays a pronounced negative region localized on the –NH_2_ group; however, this region is partially perturbed by intramolecular interactions with the –COOH group, thereby reducing effective nucleophilic availability. The meta isomer presents a more diffuse electrostatic distribution, with comparatively lower electron density concentration at the nitrogen atom. In contrast, the para isomer exhibits a well-defined negative potential localized on the amino nitrogen, resulting from more favorable resonance distribution and reduced steric interference.

It is important to note that although resonance interaction between the –NH_2_ and –COOH groups can occur in both ortho and para isomers, the net electronic effect on the amino group differs significantly. In the ortho isomer, the intramolecular hydrogen bond formed through the carbonyl oxygen modifies the overall electronic distribution within the molecule and imposes conformational constraints that indirectly affect the availability and orientation of the nitrogen lone pair for conjugation and proton-transfer processes. In addition, the strong –I (electron-withdrawing inductive) effect of the –COOH group is more pronounced at short distance, leading to a reduction in the effective electron density on the nitrogen atom. In contrast, in the para isomer, although resonance delocalization is still possible, the absence of intramolecular hydrogen bonding and the increased spatial separation reduce the direct inductive electron withdrawal. As a result, the lone pair on the amino nitrogen remains more available for nucleophilic attack, which explains its higher apparent nucleophilicity compared to the ortho isomer.

These electrostatic differences correlate with the computed activation energies and the stability of proton-transfer intermediates (SPT and DPT). Regions characterized by higher negative electrostatic potential correspond to enhanced intrinsic nucleophilicity of the –NH_2_ group. Consequently, the positional substitution pattern modulates electronic distribution and provides a rational explanation for the distinct reactivity trends observed in the condensation pathways of the three isomers. To evaluate the role of local electronic properties in governing the reactivity of the studied systems, we analyzed the molecular electrostatic potential (MEP) of the reactants. This descriptor provides insight into the electron-rich and electron-deficient regions of the molecules and is commonly used to estimate the relative nucleophilic character of reactive centers.

In this context, we investigated whether the MEP values at the amino nitrogen atom can be correlated with the activation barriers of both SPT and DPT mechanisms, with the expectation that a more negative electrostatic potential would favor nucleophilic attack and whether it would lower the corresponding energy barriers. Still, it is very likely that the overall energetic stability of the ligands may take precedence over the propensity for proton release or acceptance predicted solely on the basis of the molecular electrostatic potential.

### 3.2. Mechanistic Pathways (SPT vs. DPT)

**Figure 2:**
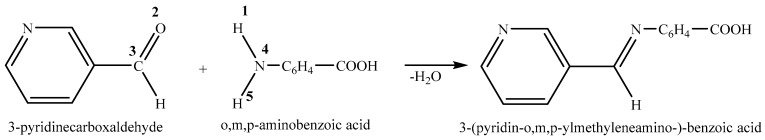
General scheme of the condensation reactions of 3-pyridinecarboxaldehyde with aminobenzoic acid isomers. The numbering of atoms participating in the condensation process is similar for all reactions.

The condensation reaction between 3-pyridinecarboxaldehyde and the three aminobenzoic acid (ABA) isomers proceeds through two alternative mechanistic pathways: a direct single proton transfer (SPT) mechanism and a solvent-assisted double proton transfer (DPT) mechanism. Both pathways were investigated in the gas phase and in methanol solution using PCM (Figure 2).

The overall condensation process leads to the formation of three corresponding Schiff base products: P1—o-(pyridin-3-ylmethyleneamino)-benzoic acid, P2—m-(pyridin-3-ylmethyleneamino)-benzoic acid, and P3—p-(pyridin-3-ylmethyleneamino)-benzoic acid. Although the final products differ only in the positional arrangement of the amino substituent, the reaction pathways and activation barriers are modulated by the electronic and geometric characteristics of each isomer, as well as by solvent participation.

#### 3.2.1. Single Proton Transfer (SPT) Pathway

In the SPT mechanism, proton transfer occurs directly from the amine group to the carbonyl oxygen of the aldehyde. This process is coupled with the nucleophilic attack of the nitrogen atom on the carbonyl carbon, leading to the formation of the C–N bond and the generation of a zwitterionic intermediate. The reaction then proceeds through intramolecular proton rearrangement and subsequent dehydration to yield the imine product. The SPT pathway involves sequential proton migration steps and is characterized by relatively high activation barriers, particularly in the gas phase, where stabilization of charge-separated transition states is limited.

#### 3.2.2. Double Proton Transfer (DPT) Pathway

In contrast, the DPT mechanism involves a cooperative two-proton relay mediated by methanol. In this pathway, the solvent molecule forms a hydrogen-bonded bridge between donor and acceptor centers, enabling simultaneous or quasi-simultaneous transfer of two protons. This relay mechanism reduces charge accumulation in the transition state and enhances stabilization through hydrogen-bond interactions. The DPT route significantly lowers the activation barrier compared to the direct SPT mechanism, especially in methanol, where dielectric screening and hydrogen-bond networks promote transition state stabilization.

**Figure 3:**
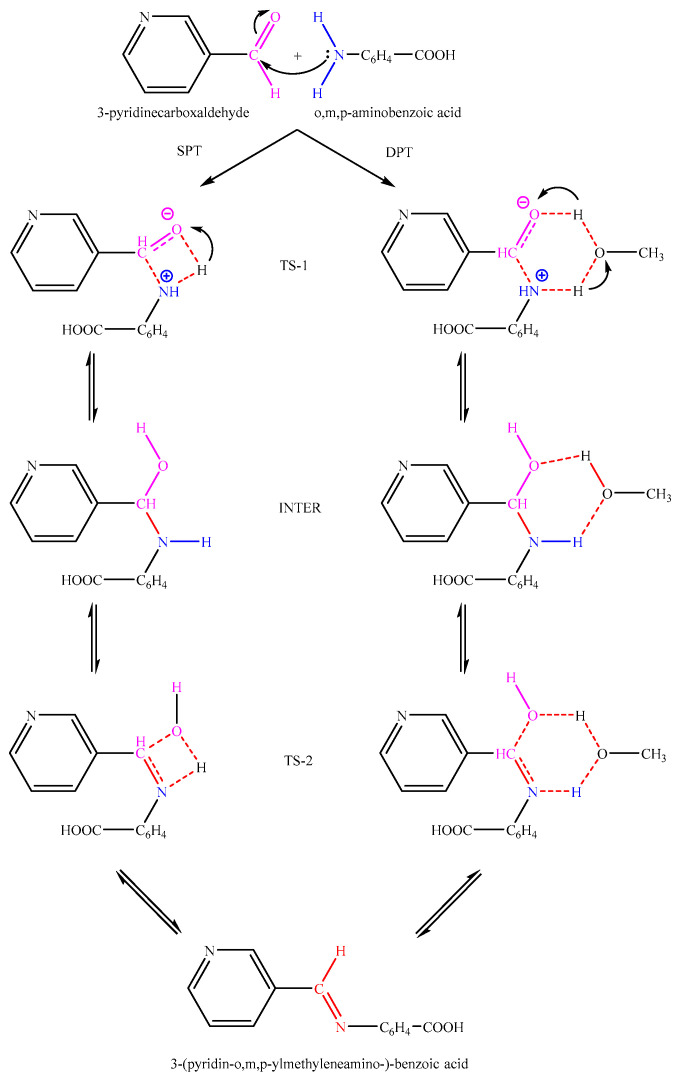
Mechanism of DPT and SPT proton transfer in the condensation reaction of 3-pyridinecarboxaldehyde with o-aminobenzoic acid.

Figure 3 illustrates the detailed reaction profile for the representative o-aminobenzoic acid system, including all stationary points and transition states identified for both SPT and DPT mechanisms.

The same fundamental mechanistic framework applies to the meta and para isomers, with differences arising primarily from electronic distribution and substituent positioning, as discussed in Section 3.1.

### 3.3. Comparative Activation Energies and Solvent Effects

The calculated activation energies for the two elementary steps (TS1 and TS2) associated with single proton transfer (SPT) and double proton transfer (DPT) mechanisms are summarized in Table 1. Calculations were performed using the B3LYP functional with both the 6-31G and 6-311++G(d,p) basis sets in order to evaluate the sensitivity of the reaction barriers (TS1 and TS2) to the level of electronic description.

#### 3.3.1. Influence of Basis Set

A consistent trend emerges from the comparison of basis sets. The 6-31G basis generally yields lower activation barriers, particularly for DPT pathways, suggesting a tendency to underestimate transition-state energies. In contrast, the extended 6-311++G(d,p) basis provides higher and more conservative barrier estimates, especially evident for the DPT mechanism of the p-isomer in the gas phase (≈24 kcal/mol at 6-31G vs. ≈43 kcal/mol at 6-311++G(d,p)).

For the SPT mechanism, differences between the two basis sets are moderate (typically within 1–3 kcal/mol), indicating the relative robustness of the calculated barriers. However, DPT pathways exhibit greater sensitivity to basis set expansion, reflecting the increased electronic complexity associated with cooperative proton transfer and hydrogen-bonding interactions.

#### 3.3.2. Gas Phase vs. Solvent Environment

In the gas phase, SPT pathways are associated with relatively high activation barriers for all isomers (≈45–55 kcal/mol), consistent with limited stabilization of charge-separated transition states. The DPT mechanism lowers these barriers substantially, particularly for the m-isomer at the 6-31G level (TS1 ≈ 14–15 kcal/mol), although larger basis sets predict more moderate reductions.

In methanol, solvent stabilization becomes evident. PCM systematically decreases activation energies for both mechanisms, with a more pronounced effect on DPT pathways. For example, in the o-isomer, DPT barriers decrease to ~23–32 kcal/mol depending on the basis set, whereas SPT barriers remain significantly higher. Overall, solvent effects contribute through dielectric screening and stabilization of polarized transition states, enhancing the feasibility of proton relay processes. However, a closer inspection of the data in Table 1 reveals that the magnitude of the solvent effect is not uniform across all cases. In certain instances, particularly for specific SPT pathways and depending on the basis set, the reduction in activation energy upon solvation is relatively small. This behavior suggests that not all transition states exhibit the same degree of charge separation or polarity, and therefore these systems benefit differently from dielectric stabilization. Consequently, while the overall trend indicates a lowering of activation barriers while in solvent, the extent of this effect is strongly dependent on the specific reaction pathway and electronic structure of the corresponding transition state.

Comparison among isomers reveals that positional substitution influences the magnitude of the activation barriers but does not alter the qualitative mechanism.

#### 3.3.3. Isomeric Effects

The ortho isomer exhibits relatively high SPT barriers, partially attributable to intramolecular interactions that restrict optimal orbital alignment. The “optimal orbital alignment” refers to the geometrical orientation that maximizes the electronic interaction between the donor lone pair (primarily on the nitrogen or oxygen atom involved in proton transfer) and the acceptor antibonding orbitals (σ* and/or π*), which is required in both SPT and DPT mechanisms. Intramolecular hydrogen bonding in the ortho isomer imposes conformational constraints that deviate the reacting orbitals from this favorable alignment, thereby reducing the efficiency of orbital overlap in both proton transfer pathways.

The meta isomer shows significant reduction of DPT barriers at the smaller basis set level, although this effect becomes less pronounced with basis set expansion.

The para isomer demonstrates enhanced sensitivity of DPT barriers to basis set choice, indicating stronger electronic delocalization effects and a more pronounced role of cooperative proton transfer.

When attempting to correlate these trends with the MEP analysis discussed in Section 3.1, the more negative electrostatic potential observed at the amino nitrogen in the para isomer compared to the meta isomer can be attributed to the more efficient electronic communication through the aromatic π-system in the para-arrangement. Although the meta isomer does not allow direct resonance interaction between the NH_2_ and COOH groups, the para geometry enables a more extended redistribution of electron density across the conjugated framework, leading to enhanced charge accumulation at the amino nitrogen. However, despite this increased local nucleophilicity, the electrostatic potential alone does not directly govern transition-state stabilization. In this context, the relative stability and structural flexibility of the isomers play a decisive role. The meta isomer, which is less affected by intramolecular interactions, exhibits greater conformational adaptability and requires a lower degree of reorganization to reach the transition state, resulting in the lowest activation barrier. The ortho isomer, although partially stabilized by intramolecular hydrogen bonding, is subject to geometric constraints that hinder an optimal arrangement during proton transfer. In contrast, the para isomer, despite exhibiting the highest intrinsic nucleophilicity according to MEP, is more electronically stabilized and requires a greater degree of charge redistribution in the transition state, leading to a higher activation barrier. While in competition, the energetic stability of the ligands should take over the possibility of releasing/accepting (the molecular electrostatic potential) a substituent (in this case, the hydrogen)—Figure 1. Thus, in the present study, the activation barrier (Table 1) controls the balance between local electronic effects and global structural and energetic factors.

Across all systems and computational levels, the DPT pathway consistently exhibits lower activation energies than the corresponding SPT route under solvent conditions. This confirms that solvent-assisted proton relay plays a decisive role in reducing energetic barriers during Schiff base formation. However, it is important to note that the intrinsically lower activation barriers of the DPT pathway compared to those of the SPT mechanism are already present in the gas phase, as shown in Table 1. This indicates that the preference for the DPT route is not solely induced by solvent effects, but is primarily governed by its cooperative mechanism, in which proton transfer occurs in a concerted manner and leads to a more efficient redistribution of charge along the reaction coordinate. In this context, the solvent acts as a secondary but reinforcing factor: while in the gas phase the energetic advantage of DPT arises from its intrinsic electronic cooperativity, in methanol the polar environment further stabilizes the highly polarized transition state, thereby amplifying the difference between DPT and SPT pathways. The cooperative hydrogen-bond network formed in methanol effectively stabilizes the developing charge distribution in the transition state, rendering the DPT mechanism kinetically more favorable.

#### 3.3.4. Detailed Mechanism—o-Isomer (Model System)

To investigate in detail the condensation mechanism between 3-pyridinecarboxaldehyde and o-aminobenzoic acid, several proton-transfer pathways were examined under both gas-phase conditions and within the PCM implicit methanol solvent model. Particular emphasis was placed on how solvent effects modulate activation energies and intermediate stabilities.

The reaction proceeds through two mechanistic possibilities: a single proton transfer (SPT) and a double proton transfer (DPT) route. In the SPT pathway, the proton from the amine group migrates directly to the carbonyl oxygen concomitantly with C–N bond formation. In the DPT pathway, methanol acts as a proton-relay agent, enabling simultaneous transfer of two protons and significantly lowering the activation barrier.

**Figure 4:**
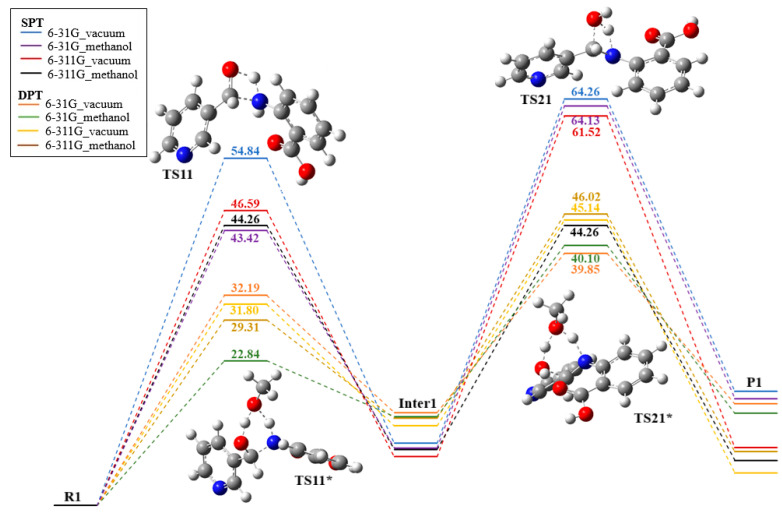
Reaction coordinate diagram for the condensation of 3-pyridinecarboxaldehyde with o-aminobenzoic acid. * Represents the transition state for double proton transfer.

Stage I—Intermolecular Proton Transfer and C–N Bond Formation. As depicted in Figure 3, the mechanism initiates with the migration of the proton H1 from the –NH_2_ group toward the carbonyl oxygen O2. The initial H1···O2 distance of ~2.08 Å progressively decreases to ~0.97 Å, marking the formation of the O–H covalent bond and completion of the proton transfer. Concomitantly, nucleophilic nitrogen N3 approaches the carbon atom C4 of the aldehyde group. The N4–C3 separation decreases from ~3.73 Å to ~1.27 Å, consistent with formation of the new C–N bond. This concerted step generates a stable intermediate (Inter1/Inter1′), whose stability is further enhanced in methanol through hydrogen bonding and dielectric screening (Figure 4).

Stage II—Intramolecular Proton Transfer and Dehydration. The intermediate subsequently undergoes an intramolecular proton transfer, in which the remaining proton H5 migrates to the carbonyl oxygen. The H5···O2 distance decreases from ~3.67 Å to ~1.00 Å, leading to the formation of the second O–H bond. This rearrangement enables water elimination and yields the final Schiff base, 3-(pyridin-o-ylmethyleneamino)-benzoic acid, whose formation is thermodynamically favored.

Methanol-Assisted Double Proton Transfer (DPT). In the DPT mechanism, methanol mediates a bridged two-proton relay, forming temporary hydrogen-bonding networks with the donor and acceptor centers. This cooperative transfer substantially decreases the barrier compared to the direct SPT process and stabilizes the associated transition states.

#### 3.3.5. Comparative Mechanistic Features of m- and p-Isomers

The condensation reactions involving the m- and p-aminobenzoic acid isomers follow the same two-stage mechanistic sequence described for the ortho derivative. Stage I consists of intermolecular proton transfer accompanied by nucleophilic C–N bond formation, while Stage II involves intramolecular proton migration and subsequent dehydration leading to Schiff base formation.

**Figure 5:**
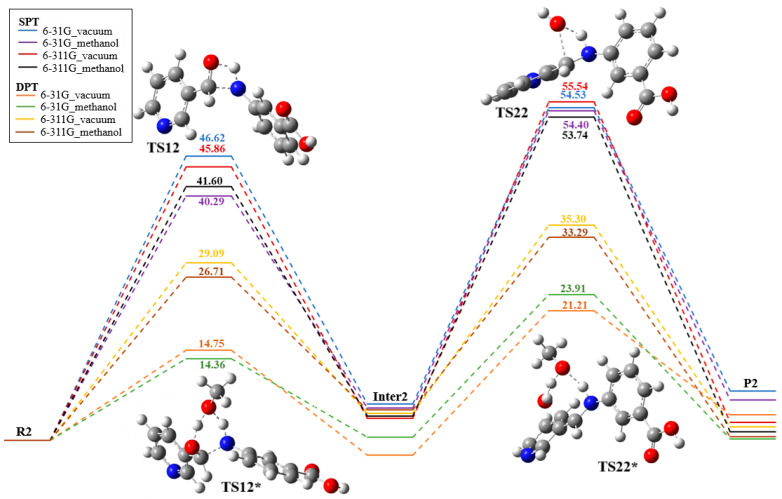
Reaction coordinate diagram for the condensation of 3-pyridinecarboxaldehyde with m-aminobenzoic acid. * Represents the transition state for double proton transfer.

Although the qualitative mechanism remains unchanged, positional substitution modulates the electronic distribution and transition-state stabilization. The meta isomer exhibits moderate variations in activation barriers without significant geometric constraints (Figure 5).

**Figure 6:**
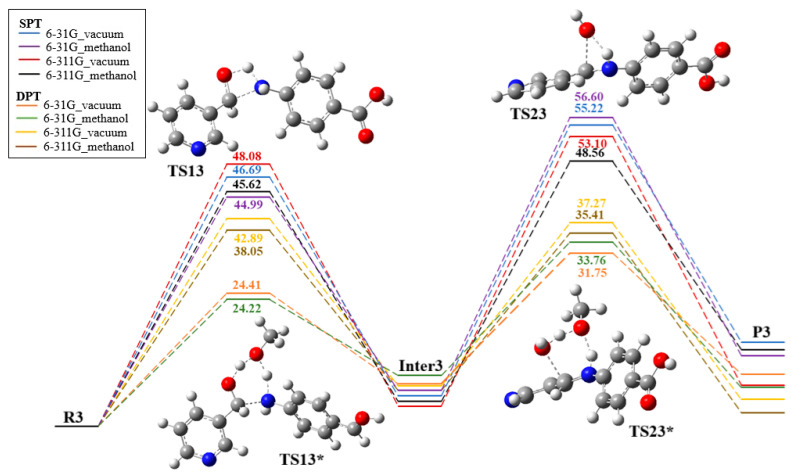
Reaction coordinate diagram for the condensation of 3-pyridinecarboxaldehyde with p-aminobenzoic acid. * Represents the transition state for double proton transfer.

In contrast, the para isomer benefits from enhanced electronic delocalization, which influences proton-transfer energetics and increases the sensitivity of DPT barriers to basis set expansion (Figure 6).

It should be noted that the energy values presented in the reaction profiles (Figure 4, Figure 5 and Figure 6) correspond to relative electronic energies of the stationary points along the reaction coordinate, whereas the activation barriers reported in Table 1 were calculated as the energy differences between the corresponding intermediates and transition states.

In all cases, the DPT pathway in methanol proceeds via a proton-relay mechanism stabilized by hydrogen bonding, resulting in lower activation barriers compared to the corresponding SPT route.

Overall, the computational investigation demonstrates that Schiff base formation between 3-pyridinecarboxaldehyde and aminobenzoic acid isomers proceeds through a two-stage proton-transfer mechanism involving C–N bond formation followed by dehydration. While both SPT and DPT pathways are mechanistically feasible, the solvent-assisted DPT route consistently provides lower activation barriers, particularly in methanol.

Overall, the activation energy values indicate that TS2 is associated with higher energetic barriers compared to TS1 for the majority of the studied systems, with only minor exceptions depending on the isomer, mechanism, and computational level. This behavior is consistently observed across both SPT and DPT pathways (Table 1). Based on these results, TS2 can be identified as the rate-determining step of the reaction mechanism, as it corresponds to the highest energy barrier along the reaction pathway and involves a more complex structural and electronic reorganization compared to TS1. However, the relatively similar TS2 barriers observed for the ortho, meta, and para isomers suggest that positional isomerism does not drastically alter the overall reaction rate. Instead, the influence of positional substitution is reflected primarily in the stabilization of intermediates, conformational flexibility, and the energetic characteristics of the preceding proton-transfer stage (TS1), leading to differences in the detailed energetic profiles while preserving comparable global kinetic behavior.

The presence of a single imaginary frequency for each transition state confirms the validity of the located saddle points and supports the proposed reaction coordinates. Solvent effects play a decisive role by stabilizing charge-separated intermediates and facilitating cooperative proton transfer via hydrogen-bond networks.

Importantly, positional substitution (ortho, meta, para) does not alter the fundamental mechanistic framework but modulates activation energies through electronic and steric effects. These findings highlight the interplay between electronic structure, solvent environment, and proton-transfer dynamics in governing Schiff base formation.

## 4. Conclusions

The present DFT study provides a detailed theoretical framework for understanding proton-transfer dynamics in Schiff base formation between 3-pyridinecarboxaldehyde and aminobenzoic acid isomers. By systematically comparing single proton transfer (SPT) and double proton transfer (DPT) pathways in both gas phase and methanol, and by evaluating the influence of basis set expansion (6-31G vs. 6-311++G(d,p)), this work offers a rigorous assessment of the electronic and environmental factors governing condensation reactivity.

The results demonstrate that Schiff base formation follows a two-step mechanism involving concerted C–N bond formation and proton migration, followed by dehydration. Both SPT and DPT mechanisms are mechanistically viable; however, a consistent kinetic preference for the DPT pathway is observed in both gas phase and solvated conditions. In the gas phase, this preference arises from the intrinsic cooperativity of the DPT mechanism, which enables a more efficient redistribution of electronic density along the reaction coordinate compared to the SPT pathway. In solution, the presence of methanol does not change this intrinsic ordering, but further enhances it by stabilizing the more polarized transition states through dielectric effects and hydrogen-bond interactions. Overall, solvent effects act as a reinforcing factor rather than the origin of the observed kinetic preference, while the fundamental driving force for the DPT mechanism remains its concerted proton-transfer character and associated electronic cooperativity.

Importantly, the sensitivity of DPT barriers to basis set expansion underscores the necessity of diffuse and polarization functions for accurately describing electron density redistribution during multi-proton transfer events. The pronounced differences observed for the para isomer further emphasize how electronic delocalization modulates transition-state stabilization and influences mechanistic preference.

Although positional substitution (ortho, meta, para) does not alter the qualitative mechanistic sequence, it significantly affects energetic profiles through subtle electronic and steric effects. These variations illustrate the delicate balance between molecular geometry, intramolecular interactions, and solvent polarization in controlling proton-transfer efficiency.

Comparison with our previous studies involving other pyridinecarboxaldehyde isomers [28,29] indicates that the position of the nitrogen atom within the pyridine ring does not significantly modify the overall reaction mechanism. In all investigated systems, both SPT and DPT pathways remain accessible, while the DPT mechanism is consistently characterized by lower activation barriers.

The present results further suggest that the activation energies are influenced more strongly by the relative energetic stability and conformational organization of the aminobenzoic acid isomers than by the position of the aldehyde group in the pyridine ring itself. In particular, differences in intramolecular stabilization and structural flexibility affect the degree of electronic and geometric reorganization required to reach the transition states, thereby modulating the corresponding activation barriers.

Beyond the specific systems investigated, this study contributes to the broader theoretical understanding of cooperative proton transfer mechanisms in organic condensation reactions. The insights gained here provide a conceptual basis for predicting reactivity trends in Schiff base synthesis and may support the rational design of functional imine derivatives in coordination chemistry, materials science, and catalysis.

## Figures and Tables

**Table 1:** The activation energies for TS1 and TS2 (kcal/mol) *.

			TS1		TS2	
			6-31G	6-311G	6-31G	6-311G
o-ABA	SPT	vacuum	54.84	46.59	54.40	54.82
		methanol	43.42	44.26	54.53	35.78
	DPT	vacuum	32.19	31.80	26.04	32.55
		methanol	**22.84**	**29.31**	**25.17**	**32.26**
m-ABA	SPT	vacuum	46.62	45.86	49.33	51.89
		methanol	40.29	41.60	48.57	49.96
	DPT	vacuum	14.75	29.09	23.66	30.42
		methanol	**14.36**	**26.71**	**23.41**	**28.33**
p-ABA	SPT	vacuum	46.69	48.08	49.70	49.45
		methanol	44.99	45.62	49.07	44.05
	DPT	vacuum	24.41	42.89	**24.03**	29.91
		methanol	**24.22**	**38.05**	25.48	**28.83**

* Bold values indicate the lowest activation energy barriers among the investigated pathways.

## Data Availability

The data presented in this study are available on request from the corresponding author.
